# Additive Manufacturing as a Solution to Challenges Associated with Heat Pipe Production

**DOI:** 10.3390/ma15041609

**Published:** 2022-02-21

**Authors:** Pawel Szymanski, Dariusz Mikielewicz

**Affiliations:** Faculty of Mechanical Engineering and Ship Technology, Gdansk University of Technology, Narutowicza 11/12, 80-233 Gdansk, Poland; dariusz.mikielewicz@pg.gda.pl

**Keywords:** heat pipe, loop heat pipe, additive manufacturing 3D printing techniques, selective laser melting, porous materials

## Abstract

The aim of this review is to present the recent developments in heat pipe production, which respond to the current technical problems related to the wide implementation of this technology. A novel approach in HP manufacturing is to utilise hi-tech additive manufacturing techniques where the most complicated geometries are fabricated layer-by-layer directly from a digital file. This technology might be a solution to various challenges that exist in HP production, i.e., (1) manufacturing of complex or unusual geometries HPs; (2) manufacturing complicated and efficient homogenous wick structures with desired porosity, uniform pore sizes, permeability, thickness and where the pores are evenly distributed; (3) manufacturing a gravity friendly wick structures; (4) high customisation and production time; (5) high costs; (6) difficulties in the integration of the HP into a unit chassis that enables direct thermal management of heated element and decrease its total thermal resistance; (7) high weight and material use of the part; (8) difficulties in sealing; (9) deformation of the flat shape HPs caused by the high pressure and uneven distribution of stress in the casing, among others.

## 1. Introduction

**Figure 1:**
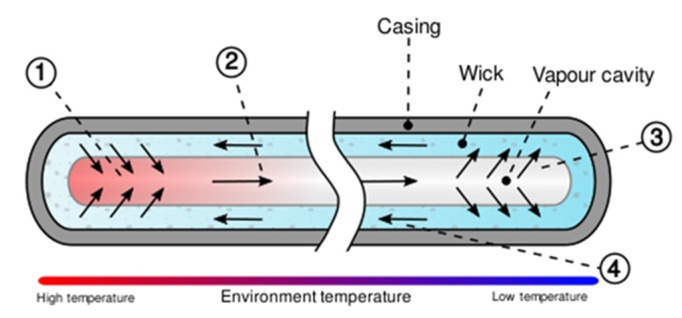
Operating principle of HP: (1) the heat from the heat source is absorbed by the liquid working fluid located inside the wick in the evaporation section, and the working fluid evaporates; (2) saturated vapour travels to the colder condenser section driven by the vapour pressure difference; (3) in the condenser, vapour releases the heat to a heat sink where it condenses back to a liquid state and is absorbed by the wick; (4) condensed liquid returns to the evaporator section through the wick structure by capillary forces [2].

Heat pipes (HPs) are highly efficient and reliable two-phase heat transfer devices that allow the transfer of the high heat fluxes over long distances or against high g-forces by evaporation and condensation of working fluid that circulates inside the device. Therefore, HP offers many advantages in comparison to conventional thermal management systems. Traditional HP consists of a sealed tube or container internally lined with a wick structure that is saturated with a quantity of working fluid, whose selection depends, i.e., on its thermophysical properties and its chemical compatibility with wick, wall material and other HP components. HPs utilise the heat of vaporisation of the working fluid circulating inside the tube to transport heat from the hot source to the heat sink and the surface tension of the working fluid to create capillary forces in the porous wick needed to circulate the fluid. Another big advantage of HPs is that the evaporator section and condenser section can be separated by a large distance allowing a small temperature difference while transferring a large amount of heat. The diagram showing traditional HP containing a wick and its operating principle is presented in Figure 1 [1,2,3].

A wide range of different configurations of HPs exist: constant and variable conductance wicked HPs, oscillating (a.k.a. pulsating) HPs, vapour chamber HPs, loop heat pipes, capillary pumped loops, rotating HPs [3,4,5,6]. HPs are designed with various shapes (e.g., flat, circular, among others) and sizes (e.g., micro) [3,5,7]. Regardless of the configuration, shape or size, the major part of HP is the porous structure (wick) is and is responsible for providing a high capillary pressure to circulate the working fluid in the device. The parameters that characterise the wicks and hence the functionality and performance of the HP are porosity, mean pore radius, permeability, wettability, effective thermal conductivity and capillary lift height ability [1,8]. These parameters are determined by the wick material properties and its internal nanostructure and mainly depend on the wick manufacturing method. Ideally, HPs require wicks generating high capillary pressure with high permeability and low thermal conductivity. HPs are commonly used as heat sinks and heat spreaders in multiple challenging fields such as security, communications, medical, test and power systems, military, consumer electronics cooling systems, aircraft and spacecraft applications, amongst others [3,4,5,6]. All aforementioned target application benefits from all the advantages of HPs (e.g., noise and vibration-free operation, passive, long-distance heat transfer, flexibility in design and assembly, compactivity, robustness, anti-gravity capability) [1,9].

Understanding the mechanisms occurring in HPs requires multidisciplinary knowledge of many complex issues, including multiphase heat transfer phenomena, thermodynamics, innovative manufacturing techniques (especially manufacturing of porous materials), material science, nanotechnology, chemistry, capillary fluid flows, fluid and solid mechanics, imaging techniques, numerical modelling and CAD and CFD techniques; hence the choice of optimal design of HP depends on various factors. The most important requirements of the HP include the amount of dissipated heat, heat transfer distance, overall thermal performance, quick start-up time, robustness, reliability of operation at anti-gravity configuration, acoustic and anti-vibration issues, manufacturing cost, weight, shape, assembly into the final appliance and the possibility of miniaturisation [10].

Despite all aforementioned advantages, numerous engineering problems and scientific challenges currently exist in the construction of state-of-the-art HPs, including:

- Difficulties of manufacturing complex, advanced and customised shapes of HPs due to limitations of traditional subtractive manufacturing processes [8,11,12,13,14,15];
- Difficulties in the integration of the HP into the electronic chassis elements or with the heat source that enable direct thermal management of the heated devices and decrease its thermal resistance. Part incorporation allows further reduction in weight or material by incorporating HP into another component [2,16,17,18,19,20];
- Difficulties in sealing the casing/wick structure. This can cause leakage and consequently failure of HP operation. This problem mainly concerns flat HPs, vapour chamber HPs and flat evaporator loop HPs with relatively long and often angular edges [16,17,21,22,23];
- Frequent deformation and ballooning of flat shape HPs, vapour chamber HPs and flat evaporator loop HPs caused by the elevated internal fluid saturation pressure. This can cause a failure of the thermal contact of the heat source and HP or a loss of the thermal junction between the wick structure and casing. The elevated saturation pressure of the working fluid may deform the envelope shape or porous structure. Such matter requires the very diligent design of the HP and might result in the increase in envelope thickness, increased HP weight or limit the choice of the working fluid [10,24,25,26];
- High customization and assembly costs and high lead time [2,21,22,23];
- Difficulties in manufacturing complicated, customised and efficient homogeneous porous structures with the desired porosity, permeability and pore radius. It can be particularly important in the production of bi-porous wick structures with enhanced thermal properties allowing HP to function against gravity. Such wicks are present in loop HP where the evaporator has wicks of different porosities and permeabilities (e.g., primary and secondary wick) and vapour passage network with different functions in loop HP evaporator [2,8,11,27];
- Difficulties in production wicks with pores evenly distributed and not randomly located. Random internal porous structure [8];
- Difficulties in manufacturing sintered wicks from specific and selected materials (e.g., aluminium). The random internal porous structure encourages heterogeneousness in the thermal behaviour of the wick [12].

Therefore, advances in the novel manufacturing techniques should be introduced to fabricate the novel and high efficient HPs that solve the above-mentioned challenges, useless room and material, and allow for higher power loads to take advantage of the passive thermal management system for electronic in multiple applications.

## 2. Additive Manufacturing in HP

**Figure 2:**
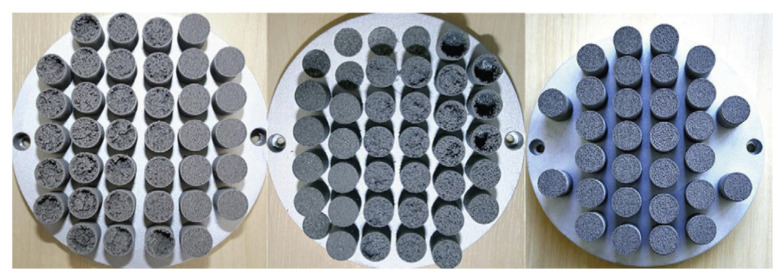
Additively manufactured porous wick samples [12].

**Figure 3:**
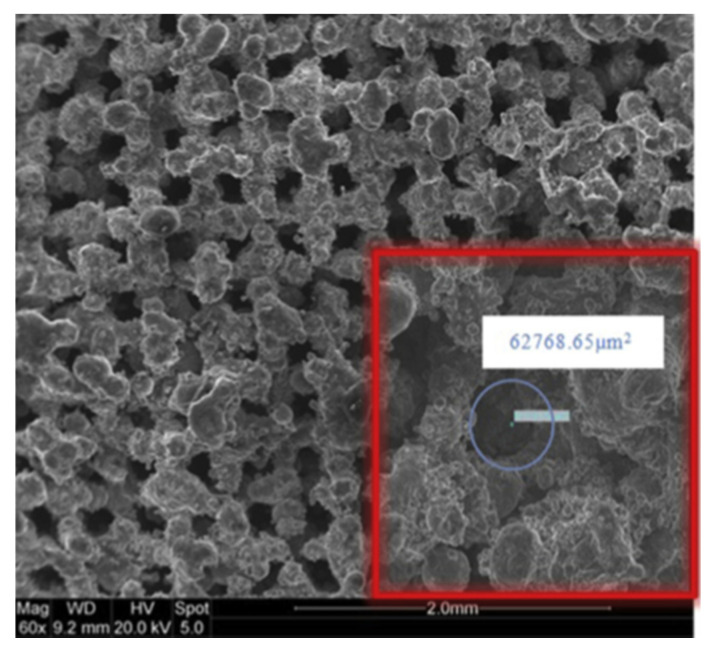
Microscopic image of AM porous structure [12].

**Figure 4:**
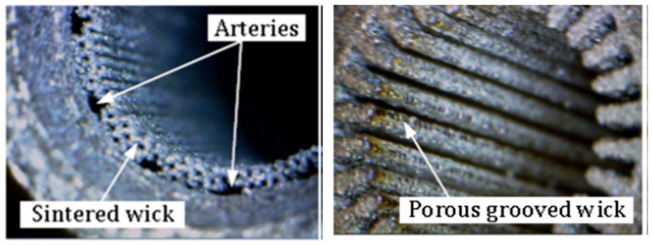
Close-up AM HPs with sintered porous wick structure: arterial wick (**left**); porous grooved wick (**right**) [4,10].

A novel approach in HP manufacturing is to utilise advanced additive manufacturing (AM) techniques where metal parts of the most complex geometries are fabricated directly from a CAD file. In 3D-printed HPs, metal structures are made in a layer-by-layer laser process that selectively melts the metal powder. This technology allows the development of an HPs with complicated or unusual geometry to maximise the interaction between the input and heat output or to maximise the surface area for evaporation and condensation phenomena. The most appropriate technology for developing HPs and their components is selective laser melting (SLM) (sometimes called laser-powder bed fusion (L-PBF)) and Binder Jetting [28]. This technology allows the manufacturing of devices with a lower cost-to-complexity ratio and quicker lean time compared to traditional manufacturing techniques and provides a unique solution for overcoming the aforementioned challenges in HP construction. The AM technique is that each layer is supported by previously printed layers underneath it; this property allows the engineer to fabricate any possible design that is difficult or even impossible to construct in traditional manufacturing techniques. Another benefit is the possibility of obtaining geometrical freedom without the limitations and constraints of other manufacturing techniques. It is also possible to highly customise the final products. The SLM process gives opportunities, especially in areas where tailored products or miniature components are required or complex parts with features unobtainable via conventional manufacturing. A detailed SLM and LPBF technology is discussed in [2,11,12,29,30,31,32,33,34]. Ameli et al. constructed the first HP with ammonia and sintered aluminium porous wick structure where the HPs were fabricated using the SLM technique with various wick characteristics. The investigation was focused on characterising the SLM HP manufacturing process and construction material of the HP. The authors proved the capability of manufacturing very complex porous structures made by sintered aluminium with different structural parameters (e.g., thickness, porosity, permeability and pore sizes) in different sections of an HP. It should be noted here that aluminium is useful HP material due to its low cost, lightweight and high strength to weight ratio [12,33]. In addition, this technology gave the possibility of manufacturing all elements of HP (e.g., porous wick, container, end cap, fill tube) in one process and one part, and hence the HP is 100% leak-free [12]. This study presented the big potential of AM technology to manufacture HPs. The view of the test samples is presented in Figure 2, a microscopic image of the sample is presented in Figure 3, and a cross-section of the AM HPs in Figure 4 [4,12].

**Figure 5:**
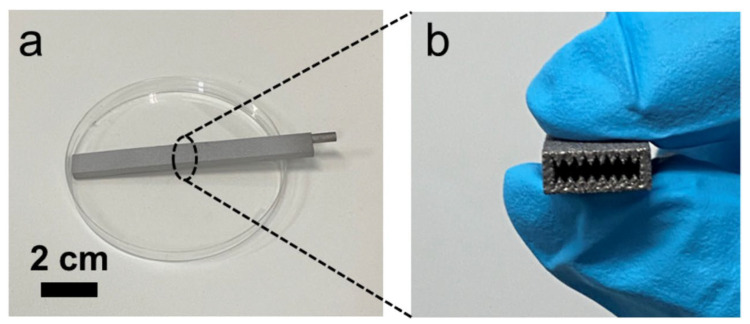
The photo of the flat miniature HP with microgrooves: (**a**) image of the AM flat HP; (**b**) cross-section of the AM flat HP [35].

The other attempt of developing a sintered aluminium heat pipe made by AM technology was made by Chang et al. [35], who designed and fabricated an aluminium flat miniature HP with microgrooves for cooling of high power LED. The dimensions of the HP were 100 mm × 10 mm × 4 mm, and their minimum thermal resistance was 0.1 °C/W and was able to transfer heat loads between 2 and 18 W. The HP had microgrooves of 0.8 mm width and 1 mm height. These micro-grooves provide a sufficient capillary force to facilitate the pumping of the working fluid back to the evaporator section. The authors noticed that the sintering process increased the roughness of the micro-grooves and inner surface of the HP, enhancing the capillary forces caused by the wick structure. Thus, the HPs heat transfer performance has become significantly improved. Moreover, this technology not only enables the creation of the complex porous wick structure but also simplifies the production of HPs. The photo of the flat miniature HP with microgrooves is presented in Figure 5.

One of the biggest engineering challenges in HP development is the aforementioned sealing casing material/porous wick structure. In most HPs assemblies, a decent seal is not always achieved. Moreover, if the knife-edge seal insertion process fails and an attempt is made to re-insert, the sintered porous wick is certainly be damaged. Such damage usually requires a wick replacement. The casing may be re-used, but a new wick must be manufactured and inserted into an envelope. It is also possible for the seal to pass verification testing after assembly but fails after acceptance tests (e.g., high-pressure proof test, burst pressure test, ageing and burn-in test or vibration test). Using AM overcomes this challenge as the HP components are made in a one-part thus, AM parts can provide a hermetic seal [21,22,23,36].

**Figure 6:**
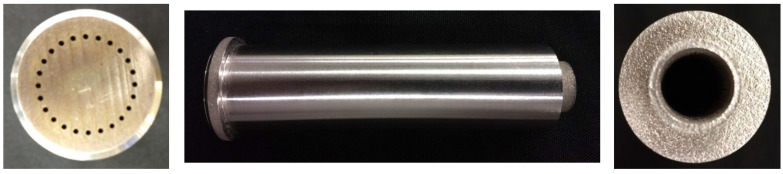
AM primary wick [21].

**Figure 7:**
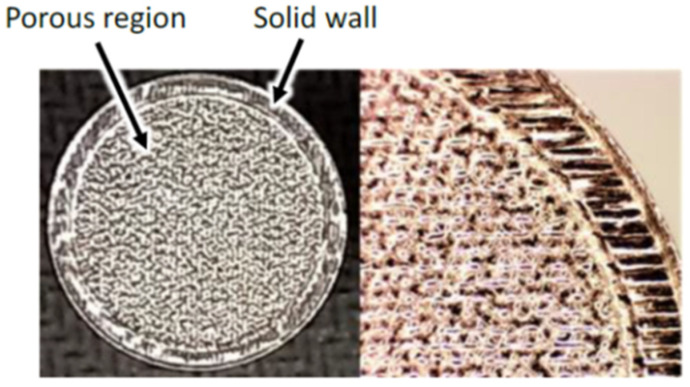
Microscopic view of AM sample featuring a porous structure surrounded by a solid wall [36].

**Figure 8:**
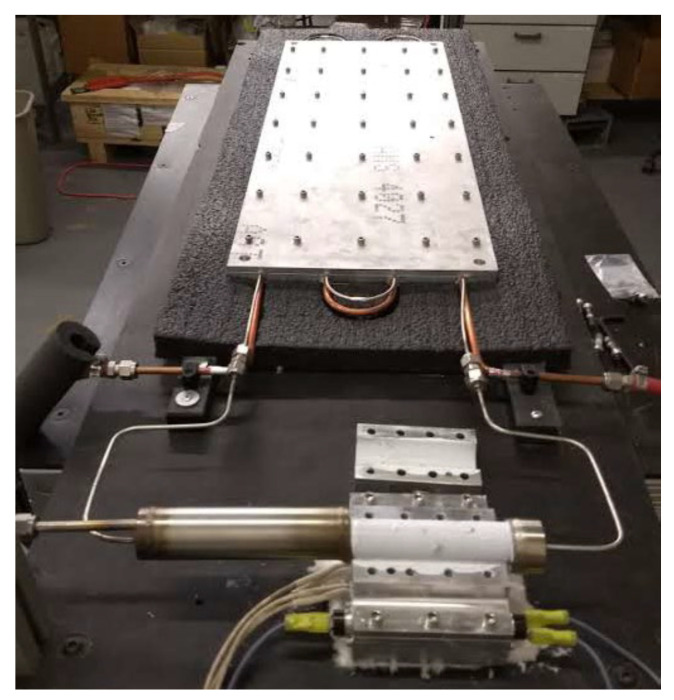
Complete LHP [21].

Loop heat pipes (LHPs) are similar devices to HPs and employ the same capillary pumping process of working fluid as used in conventional HP but operate over much longer distances and/or against gravity by deploying a primary and secondary wick that is challenging to manufacture [26,37,38,39]. Anderson et al. constructed a cylindrical LHP utilising AM method where the casing, primary porous wick and secondary wick were manufactured in a single process eliminated knife-edge seal and bimetallic joints. Such assembly drastically reduced the risk of leaks. The authors noted that eliminating secondary material such as brazing/welding fillers, which might not be compatible with the working fluid, is beneficial in LHP construction. The author constructed multiple LHPs with wicks of 9.8 µm to 125.6 µm pore diameter. In this design, the primary wick was printed with a thick envelope and then machined down to create a smooth surface for better contact with the saddle to reduce thermal resistance. The author presented AM LHP successfully and robustly operating in adverse elevation and can transfer up to 125 W for about 3.2 m [21,22,23], and in the other paper, they presented that it can operate successfully at steady-state conditions up to 350 W [36]. The LHPs were made of 316 L stainless steel. Ammonia was used as the working fluid. Moreover, the author proved that AM of LHPs can significantly reduce the overall cost of the device by eliminating expensive processes associated with multiple machining steps. The view of AM primary wick is presented in Figure 6, the microscopic view of a sample presented in Figure 7, and the view of the entire LHP is presented in Figure 8 [21,22,23].

**Figure 9:**
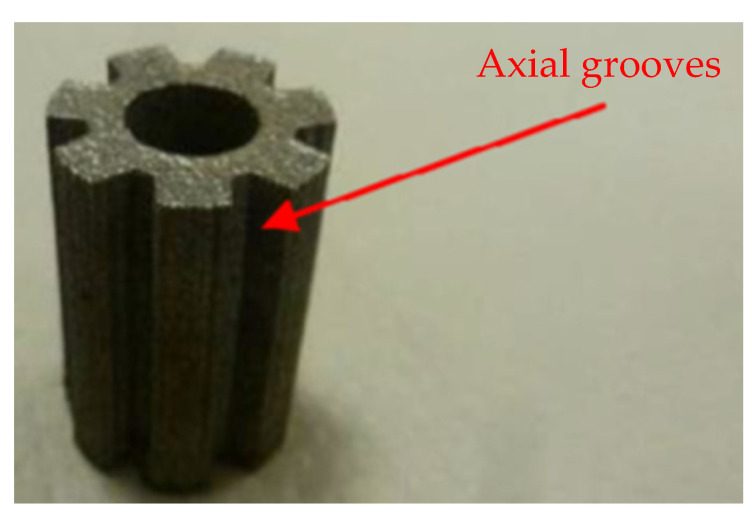
AM primary wick [8].

Sintered wicks are the most common technology for manufacturing a porous wick. However, its main drawback is the randomness and heterogeneousness of internal structure and their low mechanical strength. When we produce two identical sintered porous wicks of the same porosity, made by the same procedure and sintered at the same furnace settings, the wick and hence the overall HP performance will not be the same. The solution to this challenge can be the SLM technique, as it allows manufacturing wicks with the desired geometry, uniform pore sizes and full control over the internal structure of the porous wick where the pores are evenly distributed. The randomness of the porous structure might result in closed pores and/or irregular liquid/vapour interface, reducing its permeability and causing unsteady thermal behaviour in the HP. SLM technology provided an alternative and proven method to manufacture ultra-efficient HP’s capillary wicks generating capillary pressures matching the defined values. AM technology controls and optimises the geometry of the internal passages (e.g., porosity and its form) of the wick aiming to achieve enhanced thermo-fluidic properties and improved thermal contacts and desired design according to the specified requirements. The wick structure could be easily controlled by an AM, eliminating the randomness of the internal structure [8,39]. Estarte et al. (2017) constructed LHP with a primary wick fabricated using AM technology. This wick has a 160 µm pore diameter, and an LHP was able to transfer the heat load up to 80 W. Due to using SLM technology, an increase of about 10% in heat transfer rate was obtained compared to other similar LHPs equipped with conventionally manufactured wick structures. The external view of the AM primary wick is presented in Figure 9 [8].

**Figure 10:**
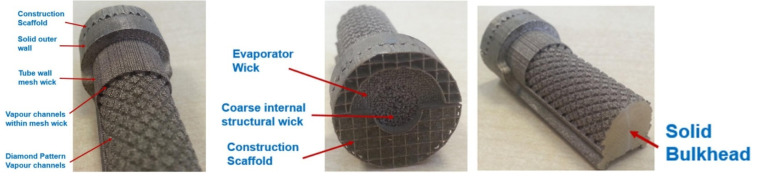
AM LHP evaporator [17].

Advancement of technology presented by Estarte et al. and Anderson et al. was presented by M^c^Glen et al. The authors presented an innovative AM LHP evaporator made by titanium alloy (Ti-6Al-4V) incorporating porous wick, solid envelope and solid internal bulkhead walls which aim to minimise the reverse flow of working fluid. The primary wick incorporates smaller pores and a secondary wick larger pores. The authors demonstrate the big potential of AM technology in wick production that can be beneficial in all capillary-driven devices. The primary wick incorporates internal vapour flow passages, which provide full coverage of the tube inside diameter with wick structure, which gives a significant performance advantage over conventionally machined wicks. The main benefit of an AM wick is in the fabrication of any form of porous structure with sophisticated geometry, which can be integrated flexibly without introducing further interfaces as a single manufactured device. This will result in a significant enhancement of the heat flux that can be dissipated. The LHP component is a good example of innovation in two-phase thermal management technology. The views of this wick are presented in Figure 10 [16,17].

**Figure 11:**
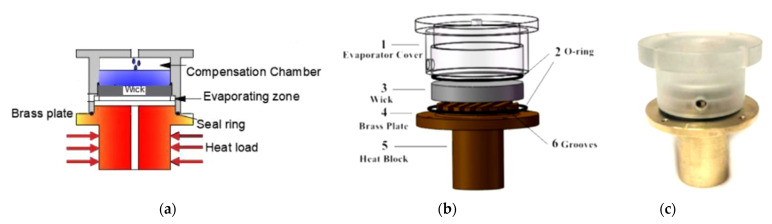
Schematic diagram (**a**,**b**) and photo of the evaporator (**c**) [39].

**Figure 12:**
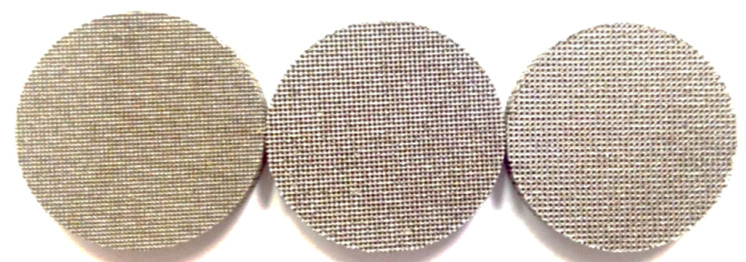
Photo of AM wicks [39].

Hu et al. constructed the flat shape LHP with the AM wick for terrestrial application. The authors demonstrated that such LHP could quickly start up (in about 100 s) at a relatively low heat load (of 20 W) and could operate stably in a wide range of heat loads from (20–160 W). According to the authors, AM wicks could break the multiple limitations of the sintered mono-porous wicks (i.e., high porosity, small pore radius, high permeability and low effective thermal conductivity simultaneously), although they constructed stainless steel wicks with pore diameters of 108–324 µm. The schematics of the evaporator and a photo are presented in Figure 11. The porous structures fabricated by AM for the needs of LHP are presented in Figure 12 [39].

**Figure 13:**
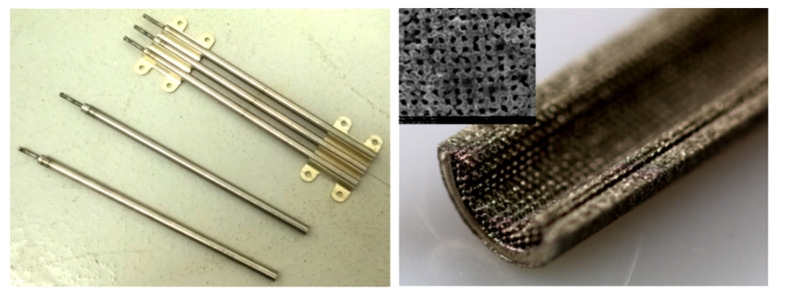
Titanium AM HP (**left**) and HP assembly (**right**) [17].

**Figure 14:**
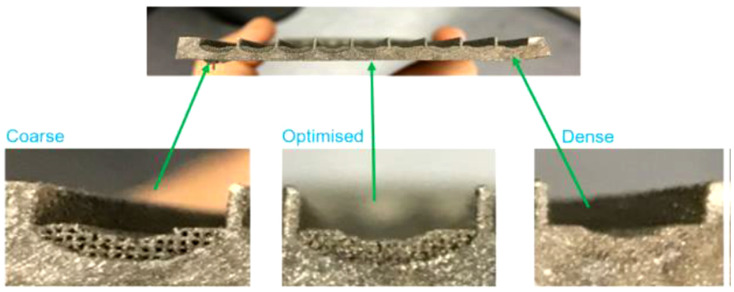
Test samples with coarse optimised and dense lattice structures [17].

The other advantage of using AM technology in HP manufacturing is the possibility of producing efficient gravity friendly wick structures that are capable of increasing the operating temperature range above that of ammonia (T_max_ > 120 °C) and overcoming the challenges faced by traditional HPs, by expanding the functionality limits when operating at adverse elevation [16,17]. Such an HP was constructed by M^c^Glen et al., who utilised AM technique to construct novel gravity-friendly titanium HPs with integrated micro- lattice capillary wick structures to enable microscale pore sizes that can function as an HP capillary wick structure. This customised HP demonstrated enhanced performance benchmarked against traditional screen mesh wicked HP and was able to transport a heat load of 30 W at elevation angles of −15° to −20° against gravity versus a maximum functional angle of −2° for traditional screen-mesh wicked HP reference test sample. The example of AM titanium HP test piece is presented in Figure 13, and the capillary wick lift height test piece is shown in Figure 14 [16,17].

**Figure 15:**
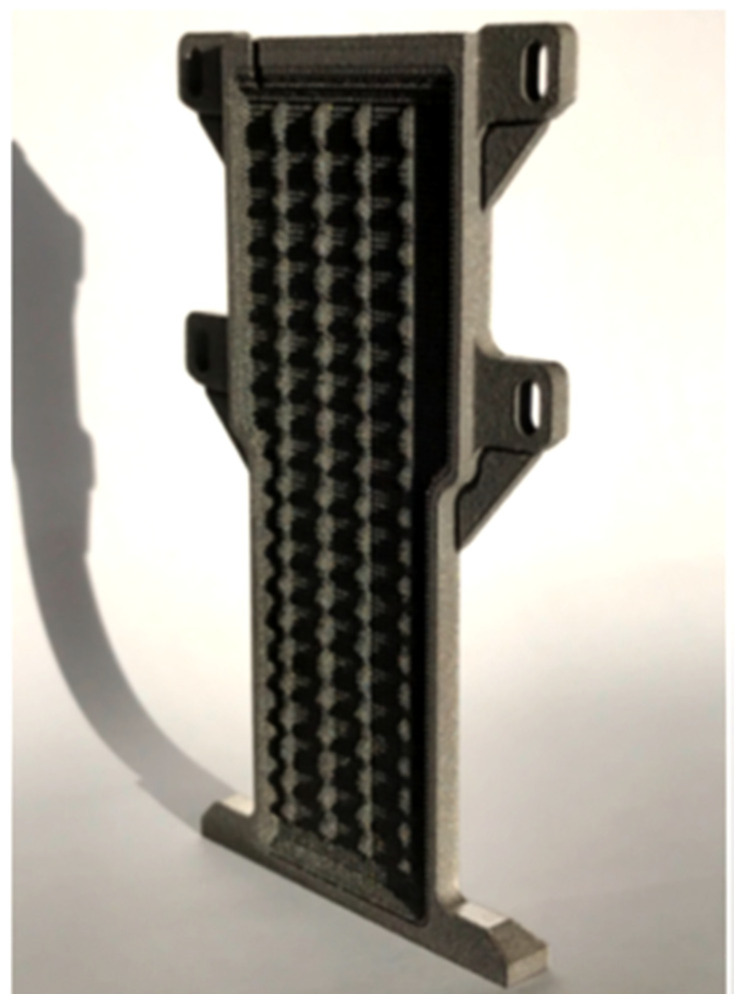
Titanium AM vapour chamber HP with integrated lattice structure [17].

**Figure 16:**
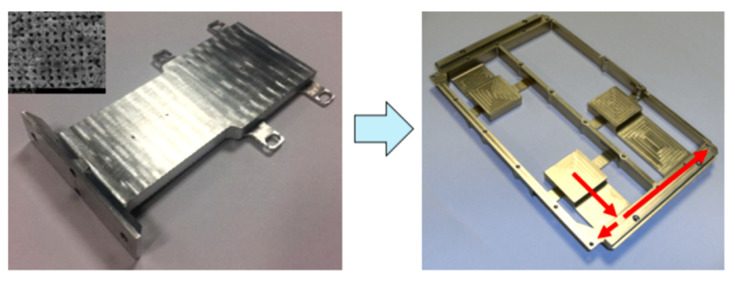
Integration of AM vapour chamber HP into the electronic unit body [17].

AM technology allows the deployment of very complex HP designs that can be combined into an electronic chassis or structural components, reducing a total number of parts and reducing a mechanical interface between HP and part and, in consequence, reducing or eliminating the contact resistance between fastened components. Moreover, this technology could integrate secondary features, such as mass and material reduction, by replacing solid walls with lattice-style walls. For example, an electronic body fulfils a dual function as a thermal conduit, without any mass penalty, which can be especially beneficial in aerospace applications [16,18,40,41] or electrical rotating machines [3,42]. M^c^Glen et al. constructed a titanium vapour chamber flat shape HP utilising LPBF technology. The view of this vapour chamber HP is shown in Figure 15, and an example of integration vapour chamber HP into electronic chassis is shown in Figure 16. The authors presented a solution where AM HP can be integrated into an electronic chassis while significantly reducing the total weight (and material utilisation) of the part. Apart from integrating a thermal management system into the unit casing and enabling direct cooling of the electronic element, titanium vapour chamber HP allows strengthening the electronic chassis and, due to its low thermal expansion, coefficient enabling increased integration with electronics. Furthermore, HPs have an elevated operating pressure which requires thick walls. This added stiffness can be beneficial in its ability to add rigidity to the overall system. Integrating components of the system is also beneficial as it streamlines the construction process making integration easier and cheaper as well as simplifying the overall design. The new technology of manufacturing vapour chambers HP might create new opportunities in application in future high-performance electronics such smartphones, laptops and tablets where low mass, compactness and tough low temperature are required.

The solution presented by M^c^Glen et al. can also be applicable in flat-shape LHP evaporators, where the deformation nature of the evaporator casing is caused by high vapour pressure inside the evaporator, which might destroy the shape of the evaporator envelope or porous wick structure. AM technology can overcome this challenge by incorporating structural elements within the evaporator and wick assembly that strengthen the device, preventing HPs’ envelope ballooning [16,17].

**Figure 17:**
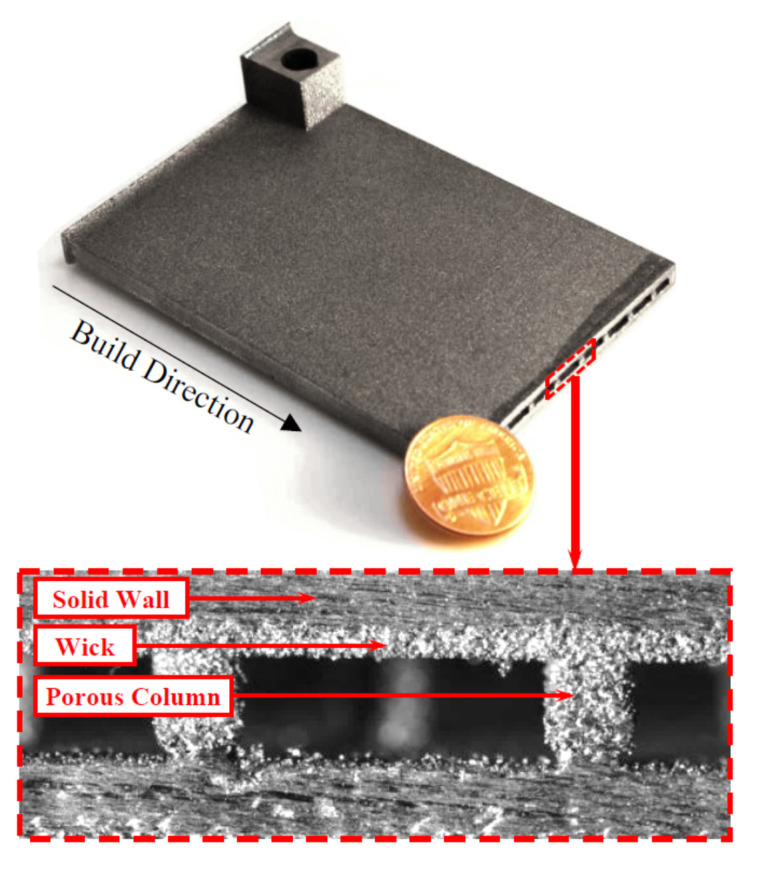
Photograph of the 3D-printed vapour chamber sample with a section cut across the width and inset view of the internal, as-printed structure [43].

Another example of successful construction of an AM manufactured vapour chamber was presented by Ozguc et al. The authors presented a capability of fabricating a micro HP of complex geometry and monolithically embedded tailored wick structure confirming the functionality of the AM technology in HP development. The vapour chamber (Figure 17) covers a 54 mm × 90 mm footprint area and, across the thickness, consists of 1 mm thick solid walls, 0.5 mm thick wicks attached on the inside of each wall and a 1.5 mm intermediate gap that serves as the vapour core. The authors proved that a relatively thick chamber wall ensures a hermetic seal of the vapour within the chamber, which is usually difficult to obtain in the traditional manufacturing methods [43].

**Figure 18:**
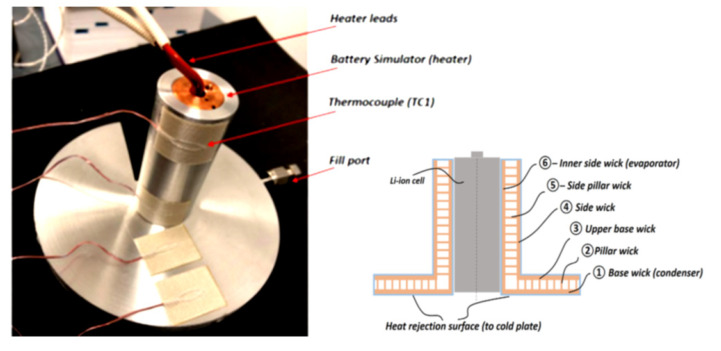
The unit cell battery case test setup (**left**) and a schematic (**right**) [18].

Additive manufacturing allows the possibility to embed porous structures and solid within one monolithic multi-functional part eliminating substantial thermal contracts resistances that exist in traditionally manufactured thermal management devices [17,18]. Therefore, Furst et al. presented a novel AM battery cell case with embedded HP. Such a case has a conductance larger than a traditional solid aluminium chassis with equal dimensions, increasing the thermal control of batteries and their robustness and preventing them from multiple dangerous effects during all phases of operation (e.g., explosion or thermal runaway). Another advantage of AM technology is reduced customisation cost and lead time of a single part. Traditional manufacturing of complex parts is costly and often generates schedule penalties, whereas AM fabrication is tenable multiple design interactions of a part. AM HP’s rapid fabrication time enables quicker time to market for new and customised design concepts. The authors presented only a concept demonstrator, and little data were available due to a patent-pending. According to the authors, the effective thermal conductivity of the cell battery HP case ranged from 316 W/mK to 1993 W/mK, and the peak conductance was much larger than the solid unit cell. The experience learned from this experiment certainly will be used in future cell battery case designs in multiple tailored thermal management devices. The photo and schematic of a prototype of battery cell chassis with embedded HP are presented in Figure 18 [18].

Thompson et al. utilised the AM technique to construct a compact mini-channel oscillating HP. The device was made of titanium alloy (Ti–6Al–4V). The utilisation of the AM method enables the construction of complex oscillating HP designs containing a casing, closed-loop and a network of circular mini-channels (Ø1.53 mm), interconnected layers and a hermetic-grade fill port. Such a complex construction can be produced with great difficulties using conventional machining methods, if at all. The authors evaluated the AM concerns related to fabrication and design limitations of titanium alloy, especially when manufacturing mini-channel embedded parts. The authors demonstrated that the system could operate effectively regardless of gravitational orientation. The AM oscillating HP operated with effective thermal conductivity of about 110 W/mK at a power input of 50 W. In parallel, the authors noted that using AM method allows for an increase in the channel density and number of layers within the device that, in consequence, improves heat transfer ratio and construction of thinner devices [44,45].

**Figure 19:**
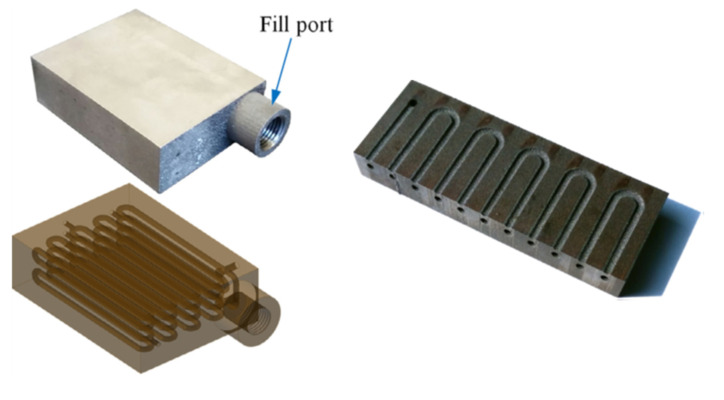
Photo of Ti–6Al–4V oscillating HP (**top left**), the transparent view of the oscillating HP (**bottom left**), photo of the sectioned prototype (**top right**) [44,45].

**Figure 20:**
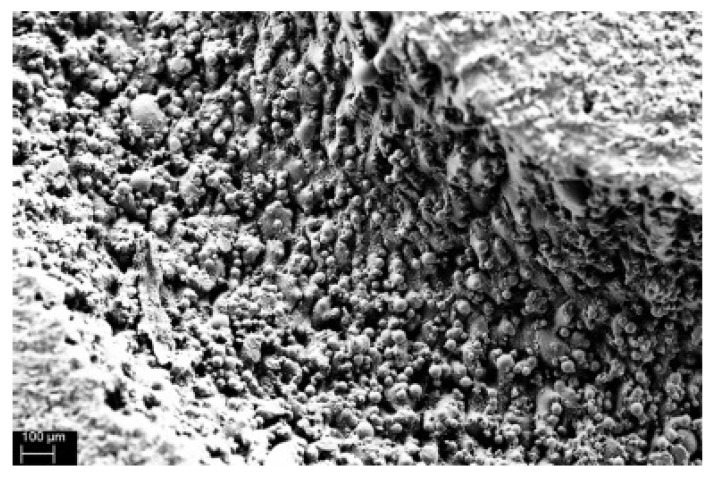
Microscope view of the internal structure of the AM oscillating HP [44,45].

Moreover, the sintered channel inner surface roughness inside the oscillating HP (a byproduct of the L-PBF manufacturing process) promotes a minimal working fluid contact angle and intensification wettability of the fluid allowing for secondary capillary forces and thin-film evaporation. These mechanisms promote boiling heat transfer during HP start-up, reduce gravity dependence, reduce start-up power and increase the oscillating HP performance. The view of oscillating HP and a photograph of a section of an oscillating heat pipe in Figure 19 and an SEM magnified image of the internal structure of the device in Figure 20. The authors concluded that due to AM technique, more complex geometries could be readily achieved, and multiple traditional thermal management systems, specifically those that use complicated mini- and micro-channels or non-circular internal tube geometries, can be re-constructed to have embedded channels with typical geometries, arrangements and surface conditions [44,45,46,47,48].

Tabulated below (Table 1) is a comparison between the most important recent developments of using AM technology in constructing wicks, HPs or LHPs.

## 3. Conclusions

AM technology takes a growing position in the HP manufacturing business, but it still needs many research activities carried out in this area. Machining and fabricating of novel 3D-printed wicks allow enhancing the limitations imposed on HPs (e.g., higher g-load resistance, longer fluid transport limit or longer life span), accelerating their adaption to higher heat fluxes and applications and filling the current “research gap” listed in the above chapter. Enhanced AM HP technologies are expected to emerge; it is hoped that in the near-to-medium term, AM can be utilised to overcome multiple challenges in HP production, such as:

- Manufacturing unconventional, advanced and tailored HPs;
- Manufacturing complicated, customised and efficient homogenous wick structures with the desired thickness, porosity, uniform pore sizes, permeability where the pores are evenly distributed and not randomly located;
- Manufacturing gravity friendly wick structures and expanding functionality limits when operating at adverse elevation;
- Quicker lean time and cost reduction due to reduction in customisation and assembly costs;
- Possibility of integration of the HP into the electronic chassis that enable direct thermal management of heated elements and decrease its thermal resistance;
- Reduction in weight (and material use) of the part;
- Solve an engineering problem with sealing casing/wick structure in HPs by eliminating knife-edge seal;
- Manufacturing wicks from specific and selected materials (e.g., sintered aluminium);
- Prevent deformation and ballooning issues caused by elevated internal vapour pressure;
- Increase the total heat flux that can be dissipated by the HP;
- Improve the start-up performance of the HP, especially at the low heat loads;

Apart from the many advantages of AM technology in HP production, this technology also has some drawbacks. The main drawback is the minimum pore size that can be manufactured using AM, which limits the use of AM, in particular, LHPs in high acceleration forces environments or long transport distance applications, where the desired pore sizes should be smaller than about 3–4 µm. However, this issue should be resolved soon with the advancement of AM technology. The second drawback of this technology is a limited choice of filament material is available for purchase. In other words, when manufacturing an HP using traditional methods, it is possible to use an almost unlimited selection of metals or alloys that can be suitable for this particular HP, but when manufacturing an HP using AM, it is possible to use only a material/firmament that your business supplier can offer or that is suitable for owned 3D printer. This issue should also be sorted soon with the advancement of AM technology, as the choice of filaments has become bigger over the years.

It is worth adding that AM technology allows the manufacturing of complex shapes of wicks or HP; however, the developments so far presented in scientific papers only present the first attempts of using AM method in HP production and have a rather basic construction. This issue should be sorted soon with the advancement of AM technology.

The other disadvantage of AM technology in HP production is the need for additional processing of AM HP. In other words, it is very difficult to predict the final mechanical parameters of the final product produced by the AM (i.e., hardness, stiffness, ductility, plasticity, strain, strength, toughness, viscoelasticity, viscosity, resistance to corrosion or cracking, etc.). The HPs require appropriate parameters to prevent deformation and ballooning caused by the internal high or vacuum pressure or unique parameters related to chemical compatibility with working fluid and material to prevent corrosion or non-condensable gasses creation. Therefore, usually, the final product (AM manufactured HP) requires additional thermal treatment (i.e., annealing, hardening, temper, etc.) before testing, which requires specialistic knowledge and additional processing.

## Figures and Tables

**Table 1:** Comparison between developments of using AM technique in manufacturing wicks, HP’s, LHPs and oscillating HPs.

Research Group	Evaporator Casing Material	Dimensions	Power [W]	Thermal Resistance [°C/W]	Wick	Heat Transport Distance	The Main Benefit of Using AM Technology in Wick, HP, LHP and Oscillating HPs Manufacturing
Ameli et al., 2013 [12]	Aluminium	Ø14 mm × L70 mm	N/A	N/A *	Aluminium Pore radius 300 µm and 500 µm	70 mm	Possibility of manufacturing an aluminium sinter-style porous wick structure in one process with machining wall, end cap and fill tube.
Thompson et al., 2015 [46]	Titanium alloy Ti–6Al–4V	Flat plate 50.8 mm × 38.1 mm × 15.8 mm	5–50	0.32	Pore radius 15–45 µm	50.8 mm	A significant increase in thermal conductivity; Surface roughness inside the device can increase the capillary pumping power of the oscillating HP and boost boiling heat transfer during start-up.
Esarte et al., 2017 [8]	Copper	Volume 2827 mm^3^ Active length 23.2 mm	57–120	0.15	Stainless steel Pore radius 80 µm	100 mm	Improved the geometric size of the internal wick passages, intending to achieve an optimal design according to the desired requirements.
Anderson et al., 2017–2021 [21,22,23]	Stainless steel	Ø25.4 mm × L10.16 mm	5–350	0.13	Stainless steel Pore radius 4.9 µm	N/A	Improvement in operation at low powers, against gravity, during rapid changes in heat input power; Much cheaper than traditional LHP fabrication techniques; Eliminates the knife-edge seal to improve reliability.
Hu et al., 2020 [40]	Stainless steel	Flat dishØ 30 mm × H5 mm	20–160	0.031	Stainless steel Pore radius 100 µm	150 mm	Boost start-up; Lowering the evaporator wall temperature during the operation.
M^c^Glen et. al., 2020 [17]	Titanium	Ø8 mm × L200 mm	20–30	N/A	Titanium Pore radius 100 µm	200 mm	Enhancing performance of the HP operation against gravity.
Jafari et al., 2020 [13]	Stainless steel	Flat dish 40 mm × 20 mm × 6 mm	0.15–16.5	N/A	Stainless steel Pore radius 216 µm	40 mm	Improvement of HP performances compared to conventional wick structures; Increase in the evaporating meniscus density at the liquid-vapour interface due to the presence of sintered powder features; Allowing thermal management systems to operate with lower temperature differences between the hot and cold interfaces with an identical operating temperature.
Furst et al., 2020 [18]	Aluminium alloy AlSi10Mg	Cell Battery case	60	0.037	Pore radius 51 µm	72.4 mm	Reduction in customisation cost and lead time of a single part; Enabling complex mechanical designs and incorporating structural elements of thermal management system into the electronic casing and enable direct heat control of the heated devices.

* N/A–not applicable.

## Data Availability

The data reported and analysed in this paper has been found on the referenced papers, documents and books. All the copyright permissions to use the figures published in the manuscript has been received.
